# Subtyping Obsessive-Compulsive Disorder: Neuropsychological Correlates

**DOI:** 10.1155/2003/782718

**Published:** 2003-01-01

**Authors:** Catherine L. Harris, Wayne M. Dinn

**Affiliations:** Department of PsychologyBrainBehavior & Cognition ProgramBoston UniversityBostonMAUSA

**Keywords:** obsessive-compulsive disorder, schizotypal personality, neuropsychological, orbitofrontal

## Abstract

We administered neuropsychological measures considered sensitive to prefrontal dysfunction (both orbitofrontal and dorsolateral prefrontal neocortex) to obsessive-compulsive disorder (OCD) patients and control subjects. OCD subjects exhibited performance deficits, in comparison to community controls, on three measures sensitive to orbitofrontal neocortex dysfunction. Contrary to expectation, OCD patients also exhibited performance deficits on measures sensitive to dorsolateral prefrontal neocortex dysfunction. However, distinct neurocognitive profiles emerged when we examined the impact of comorbid schizotypal personality features on neuropsychological test performance. Primary OCD patients displayed impaired performance on measures sensitive to orbitofrontal dysfunction; however, they did not differ from control subjects on tests of dorsolateral function. OCD subjects presenting with schizotypal personality features performed poorly not only on tests sensitive to orbitofrontal dysfunction, but also on tests sensitive to dorsolateral dysfunction. Findings suggest that OCD can be subdivided into clinical subtypes, and distinct prefrontal subsystems may be differentially involved in these subtypes.

## 1. Introduction

Converging lines of evidence suggest that dysfunction of a cortical-subcortical circuit involving the orbitofrontal region underlies obsessive-compulsive disorder (OCD). Functional neuroimaging studies implicate the orbitofrontal system. Significant elevations in glucose utilization and regional cerebral blood flow have been observed in both orbitofrontal and caudate regions of OCD subjects in comparison to controls [1–4]. Imaging studies have also documented increased glucose utilization and regional cerebral blood flow (rCBF) in orbitofrontal circuit structures during symptom provocation among OCD subjects [5–8]. Neuropsychological testing has also revealed a pattern of neurocognitive impairment among OCD patients which implicates the orbitofrontal region. OCD patients demonstrate a greater degree of impairment on neuropsychological measures considered sensitive to orbitofrontal dysfunction in comparison to psychiatric and non-clinical controls [9–11]. Abbruzzese and colleagues reported that OCD subjects exhibited performance deficits on the Object Alternation Test in comparison to schizophrenic and normal control subjects [9,10]. Poor performance on the Object Alternation Test is associated with orbitofrontal dysfunction in human and non-human primate subjects [10,12–16]. In several studies, OCD patients did not demonstrate performance deficits on the Wisconsin Card Sorting Test (WCST) [9,10,17]. Impaired performance on the WCST is associated with prefrontal (particularly dorsolateral-prefrontal) dysfunction. Neuropsychological test findings and functional neuroimaging studies suggest that a highly select deficit involving the orbitofrontal system underlies OCD [10,11].

Researchers have come to appreciate that the prefrontal region is not a unitary structure; rather, it is fractionable into distinct subsystems which maintain extensive reciprocal connections with other cortical and subcortical structures. An extensive body of evidence suggests that dorsolateral-prefrontal cortex (DLPFC) mediates executive functions [18,19], while the or-

ISSN 0953-4180/03/$8.00 © 2003 – IOS Press. All rights reserved bitofrontal system plays a significant role in the processing of emotional information and inhibitory control, and modulates sensitivity to reinforcement contingencies [20]. Following prior work in the neuropsychiatric literature, we assume that orbitofrontal cortex mediates sensitivity to dynamically changing reinforcement contingencies, and thus may be particularly important for modulating individuals' response to the social world and other threat-laden situations.

### 1.1. Conflicting findings

Conflicting results have arisen regarding performance on measures of executive function. As noted previously, several studies found that OCD patients did not demonstrate performance deficits on tests assessing executive functioning [9,10,17,21–23,25,26]. However, Lucey et al. [27] reported that 19 OCD patients demonstrated significant performance deficits on the WCST in comparison to matched controls. Indeed, a number of studies found that OCD patients showed greater neuropsychological deficits on tests of executive function [24,27–33]. How can we account for these conflicting findings? One possibility is that executive function deficits among OCD patients are associated with the presence of comorbid psychiatric conditions. In prior work, we observed that OCD patients who did poorly on executive function tasks had high schizotypy scores. Does the proportion of OCD subjects presenting with schizotypal personality features account, at least in part, for the conflicting findings in the neuropsychological literature? A significant subset of OCD patients display schizotypal personality traits. Stanley et al. [34] reported that 28% of an OCD sample exhibited schizotypal features; however, only 8% met diagnostic criteria for SPD. Mavissakalian et al. [35] found that 16% of an OCD patient group met DSM-III criteria for SPD, while Baer and Minichiello [36] reported that 35% of a series of patients with OCD seeking treatment at an OCD clinic presented with comorbid SPD.

In the present study, we examined the neuropsychological profiles of OCD patients presenting with similar patterns of comorbidity and medication use, while differing in the degree of schizotypal personality features present. We predicted that OCD patients presenting with schizotypal features would exhibit deficits, relative to primary OCD subjects, on tests assessing executive function. This prediction was based on studies which found that individuals receiving a SPD diagnosis or university students psychometrically defined as schizotypic demonstrate a greater degree of impairment on tests of executive function [37–45]. Schizotypic subjects also exhibit performance deficits on visuospatial working memory tasks [46–48]. A subset of OCD patients display schizotypal personality traits and the presence of schizotypal personality disorder (SPD) is a strong predictor of poor treatment outcome [49–51]. However, there is a surprising dearth of research examining the clinical presentation and neuropsychological test performance of patients presenting with OCD and comorbid SPD.

### 1.2. Research goals

Our principal research objectives were: 1) to investigate the claim that OCD subjects demonstrate a selective neuropsychological deficit involving the orbitofrontal system; and 2) to determine whether OCD patients presenting with and without prominent schizotypal personality features demonstrate distinct neurocognitive profiles.

## 2. Method

Neuropsychological measures considered sensitive to prefrontal dysfunction were administered to OCD patients ($n = 25$) and control subjects ($n = 11$) recruited from the general population. The OCD group comprised 13 female and 12 male subjects. Their ages ranged from 20 to 68 years ($M = 39.4$; $SD = 14.5$). Five patients were recruited from a private psychiatric facility and met Diagnostic and Statistical Manual of Mental Disorders (4th ed.) (DSM-IV) diagnostic criteria for OCD [52]. Diagnoses were made by a board-certified psychiatrist or a licensed clinical psychologist specializing in the treatment of anxiety disorders. We also recruited individuals with OCD ($n = 20$) from the general population. This was done via newspaper advertisements, seeking individuals diagnosed with OCD, in the health/science section of a major metropolitan newspaper. The mean educational level of the OCD group was 15.6 years ($SD = 1.6$).

Twenty-three OCD subjects were right-handed and two subjects were left-handed, as determined by self-report. We conducted semi-structured interviews and employed the Mini International Neuropsychiatric Interview [53] to identify comorbid psychiatric conditions. The MINI is a brief structured psychiatric interview based on DSM-IV and ICD-10 diagnostic criteria. The MINI provides diagnostic algorithms and can be administered by clinicians or trained lay interviewers [53]. A pre-testing interview elicited information regarding treatment and medical history, educational level, history of traumatic head injury or central nervous system pathology, vision or hearing difficulties, and handedness. Each patient provided the name and institutional affiliation of the clinician who established the OCD diagnosis. The majority of subjects were assessed at OCD specialty clinics. The remaining subjects received an OCD diagnosis from a licensed clinical psychologist or a board-certified psychiatrist specializing in the treatment of anxiety disorders. OCD patients exceeded the symptom threshold on the MINI (OCD module). OCD subjects meeting DSM-IV diagnostic criteria for the following Axis-I syndromes were excluded: major depressive episode; bipolar disorder; psychotic disorders; substance abuse/dependence disorders (within the past year). Individuals were also excluded if they reported a history of electroconvulsive treatment or traumatic head injury (with loss of consciousness and cognitive sequelae). All subjects were free of severe or acute medical illnesses that may impair neurocognitive function. No other exclusionary criteria were used because we were interested in obtaining a naturalistic sample. We noted if the participant was undergoing pharmacotherapy at the time of testing, but did not exclude subjects on the basis of medication use. To address the issue of medication effects on neuropsychological test performance, we compared the neurocognitive profiles of medicated and unmedicated OCD patients (as discussed in the results section).

Eleven control subjects were recruited from the general population. Their ages ranged from 21 to 47 ($M = 35.4$; $SD = 9.9$). The mean educational level of the control group was 16.1 years ($SD = 2.3$). Ten control subjects were right-handed and one subject was left-handed as determined by self-report. The control group comprised 6 female and 5 male subjects. Participants responded to flyers posted in the community seeking individuals interested in participating in a study examining the neuropsychology of personality. OCD subjects and participants recruited from the community received financial compensation ($25). Written informed consent was obtained from all participants.

### 2.1. Protocol

The neuropsychological test battery included three tasks believed to be sensitive to orbitofrontal dysfunction: the Object Alternation Test [12], Stroop Color-Word Test, and a visual Go/No-Go discrimination task [54]. Poor performance on the Object Alternation Test may be indicative of orbitofrontal dysfunction [12–16]. Difficulty inhibiting a prepotent response during the Stroop and Go/No-Go tasks may also indicate ventral/orbitofrontal dysfunction. Subjects undergoing positron emission tomography (PET) while participating in the Stroop Color-Word Test demonstrated right orbitofrontal activation as well as increased activity in bilateral parietal structures [55]. During a second experiment, Bench et al. [55] documented right frontal polar and right anterior cingulate activation during Stroop task performance. Efficient performance on the Stroop task requires sustained attention and impulse control. Therefore, the Stroop Color-Word Test should be considered a broadly frontal task. Nevertheless, orbitofrontal or ventral prefrontal systems may be implicated when presenting symptoms include impaired performance on neurocognitive tasks which require the subject to suppress a prepotent response pattern. Several studies demonstrated that performance deficits among human and non-human primates on Go/No-Go tasks (e.g., RT slowing and frequency of false alarms) were associated with orbitofrontal and ventral prefontral lesions [56,57]. Rubia and colleagues [58] documented increased activation in mesial frontal and inferior frontal cortex, and caudate nucleus during a Stop Task among healthy adult subjects. The Stop Task is similar to the Go/No-Go Task (i.e., conflict blocks) used in the present study. Findings suggest that the Go/No-Go Task should be considered a broadly frontal task.

Tests of executive function and verbal fluency included the Controlled Word Fluency Test (FAS Test) [59], Porteus Maze Task [60], Trail-Making Test (Parts A & B) [61], and Divergent Thinking Task [62] (based on Guilford and Hoepfner). Performance deficits on tests assessing executive functions are believed to reflect dorsolateral- or mesial-prefrontal dysfunction. Impaired performance on verbal fluency and divergent thinking tasks may reflect dorsolateral-prefrontal dysfunction. Regional cerebral blood flow (rCBF) studies revealed significant flow augmentation in dorsolateral-prefrontal cortex during verbal fluency tasks [63,64] and classical tests of frontal executive function. An extensive body of evidence suggests that DLPF cortex mediates executive functions (e.g., planning, organization, and keeping in mind diverse future consequences). Moreover, patients with damage to the dorsolateral aspect of the prefrontal region display performance deficits on classical tests of frontal executive functioning. The Trail-making Test (Parts A & B) taps visuospatial scanning and graphomotor skills. Efficient

Trails B performance depends upon the ability to maintain information on-line and shift cognitive set. Efficient performance on verbal frequency tasks requires efficient organizational strategies.

Several lines of research suggest that many of the experimental tasks employed in this study possess localizing value. Research suggests that DLPF cortex mediates executive functions, while orbitofrontal cortex modulates sensitivity to reinforcement contingencies and plays a major role in behavioral inhibition. The imaging and human lesion literature is broadly consistent with the notion that the prefrontal region is fractionable into functionally distinct subsystems and that specific neurocognitive tasks may tap specific prefrontal subsystems. However, our findings must be interpreted with caution. It is important to bear in mind that neurocognitive tests are only indirect measures of neurophysiological function and the localizing value of such tasks is uncertain. All computerized tasks were administered on a Macintosh IIci. Reaction time and voice onset latencies were collected using a millisecond timer that interfaces with PsyScope, experimental design software developed by Cohen and colleagues [65]. Neurocognitive tests were administered by research technicians who followed a standardized testing protocol. Tasks were administered in the same order for all subjects and technicians were not blind to the clinical status of participants.

### 2.2. Description of neurocognitive tests

#### 2.2.1. Object Alternation Test (OAT) (computer version based on Freedman) [12]

Subjects viewed two distinct stimulus objects (red cup and blue cup) on a computer monitor. During each trial, the computer "hides" a coin in one of the cups and subjects must determine which cup contains the coin. The coin moves to the unoccupied cup following a correct response. Subjects received immediate feedback from the computer regarding accuracy of response following each choice. The message "You are right" or "You are wrong" is displayed following each choice. Performance on the Object Alternation Test was determined by number of trials required to induce the solution. Participants reach criterion when they correctly predicted coin location on 12 consecutive trials. Subjects' score was the trial number of their last wrong response before the onset of their run of 12 correct trials. A low score thus indicates rapid induction of the rule governing the coin's position. The score of participants who never induced the solution was set to 50, the maximum number of trials. A drawback of this version of the OAT is that the red and blue cups always remain in the same location on the screen. Participants can focus their attention on movement of the coin between spatial locations rather than between objects (thus, the task is a spatial alternation task). Work with non-human primate subjects suggests that spatial cues are processed by the dorsal visual stream, while information about objects is processed by the ventral stream. It is controversial whether these streams extend to prefrontal cortex, however, if they do, the implication is that orbitofrontal/ventral cortex would be more specialized for tracking object identity, while DLPFC would be more sensitive to spatial cues. Given that these prefrontal subsystems may be specialized for processing spatial vs. object cues, we modified the OAT so that cup location was random: the red cup and the blue cup would appear at random locations across the computer screen (Object Alternation Test-Modified Version [OAT-MV]). Deacon (personal communication, 1999) suggested that the removal of spatial cues would render the modified version more sensitive to orbitofrontal dysfunction. After administering the Object Alternation Test to 10 OCD patients and 11 community control subjects, we modified the task. We administered the modified version (OAT-MV) to seven primary OCD patients and eight OCD/SP subjects.

#### 2.2.2. Stroop Color-Word Test

We developed a computer version of the Stroop Color-Word Test. Participants were instructed to read words describing colors as rapidly as possible. Words were displayed one at a time on the computer monitor, with the ink color being either consistent or inconsistent with the given word. During the first block (non-conflict block) the subject was asked to read the word displayed on the monitor and ignore the ink color (40 trials). During the second block (conflict block) the participant was asked to identify the ink color (40 trials). Response latencies were recorded using a voice-activated millisecond timer. The dependent measure was response time.

The experimenter sat next to the subject and pressed computer keys to record whether the subject had correctly read the word (block 1) or correctly named the ink color (block 2).

#### 2.2.3. Go/No-Go Task–based on Lapierre et al. [54]

Subjects press the space bar as quickly as possible when a $2 \times 2$ cm blue square appears (against a white background) on a computer monitor. During the first block (50 trials) only blue squares were displayed. During the second block (50 trials) subjects were instructed to respond when the blue square appears and refrain from responding when a $2 \times 2$ cm blue cross is displayed. During the third block (50 trials) subjects were instructed to respond when the blue cross is displayed and refrain from responding when the square appears. The blue square or blue cross appear at random locations across the computer screen. The interstimulus interval was also randomized with intervals of 100, 250, 400, 500, 750, 1000, or 2000 milliseconds.

*2.2.4. Verbal Fluency Test (FAS test) [59]*

During the Word Fluency Test the subject was asked to write down as many words as possible that begin with a specific letter (F, A, or S) during three one-minute trials. Total number of words produced was the dependent measure. Participants received the following instructions based on Spreen and Strauss [70]:

> The experimenter will say a letter of the alphabet. Please write down as many words that begin with that letter as quickly as you can. For instance, if the experimenter says 'B', you might write bad, battle, boxing, and so on. Do not include words that are proper names such as 'Boston' or 'Bob' or the same word again with a different ending such as 'run' and running.'

*2.2.5. Divergent Thinking Task (DVT) – based on Guilford and Hoepfner [62]*

During this task subjects were asked to name as many different uses of a newspaper as possible during a one-minute trial. They were provided with the following example: one use is rolling up the newspaper to swat a mosquito. Number of alternate uses was the dependent measure.

We administered two additional tests of executive function to OCD subjects, the Porteus Maze Task [60] and the Trail-Making Test (Parts A & B) [61]. We did not administer these additional measures to community control subjects. We directly compared the neurocognitive profiles of OCD patients presenting with and without prominent schizotypal personality features.

*2.2.6. Porteus maze task [60]*

Participants were required to find the exit route from a relatively complex maze. Efficient performance requires planning and anticipation of blocked routes. Time to completion was the dependent measure.

*2.2.7. Trail-Making Test (Parts A and B) [61]*

During Part A of the Trail-Making Test subjects were instructed to connect 25 numbered circles (1–25) randomly distributed over an $8 \times 11$ sheet of paper. Subjects were instructed to connect circles as rapidly as possible. During Part B subjects were required to connect 25 circles which contain numbers (1–13) or letters (A–L) and must sequentially alternate between numbers and letters (that is, 1-A-2-B-3-C, and so forth). Subjects received feedback when circles were connected out of order. Time to completion was the dependent measure.

OCD subjects also completed the Frontal Lobe Personality Scale (FLPS-Patient Version) [66], the Schizotypal Personality Questionnaire-B(SPQ-B) [67], and the Limbic System Checklist-33 (LSCL-33) [68]. We did not administer these additional scales to community control subjects.

## *2.3. Clinical scales and personality questionnaires*

*2.3.1. Mini International Neuropsychiatric Interview (MINI) [53]*

The MINI is a brief structured psychiatric interview based on DSM-IV diagnostic criteria. The MINI provides diagnostic algorithms and can be administered by clinicians or trained lay interviewers.

*2.3.2. Frontal Lobe Personality Scale (FLPS-patient version) [66]*

Respondents were instructed to indicate how frequently they experience symptoms or exhibit behaviors associated with frontal lobe syndromes including: 1) behavioral disinhibition; 2) executive function deficits; and 3) apathy, reflecting orbitofrontal, dorsolateral-prefrontal, and mesial-prefrontal/anterior cingulate dysfunction, respectively [66].

*2.3.3. Schizotypal Personality Questionnaire-B (SPQ-B) [67]*

The SPQ-B is a 22-question, self-report measure used to screen respondents for the presence of schizotypal personality features. The SPQ-B yields a total score and three subscale scores reflecting: 1) Cognitive or Perceptual Distortions; 2) Interpersonal Deficits; and 3) Disorganization. Scores range from 0–22. Items correspond to DSM-IV diagnostic criteria for schizotypal personality disorder. The SPQ-B is a psychometrically sound instrument which compares favorably to established measures of schizotypal personality.

*2.3.4. Limbic System Checklist-33 (LSCL-33) [68]*

The Limbic System Checklist is a 33-item symptom inventory. Respondents were instructed to indicate how frequently they experience symptoms associated with temporolimbic dysfunction including "paroxysmal somatic disturbances, brief hallucinatory events, visual disturbances, automatisms, and dissociative disturbances" [68, pp. 302].

Time to completion of the protocol was highly variable across clinical subjects. In some cases the experimental session had to be concluded early due to subject fatigue; in other cases subjects asked to end specific tasks before they were completed. For these reasons not all tasks were run on all subjects (see Table notes). In our analyses, degrees of freedom in the t statistics vary according to how many subjects participated in that task.

*2.4. Data analysis plan*

Independent t-tests (two-tailed) were conducted. Results are presented in Tables 1–4. Effect sizes (omega $^2$) were calculated to ascertain the strength of group differences. To determine if distinct neurocognitive profiles were associated with the presence of schizotypal personality features, we classified OCD subjects in the following manner and conducted separate analyses: OCD patients exceeding a cutoff score of 12 on the SPQ-B were categorized as OCD/SP (OCD patients presenting with schizotypal features). The mean SPQ-B score for the OCD/SP sample was 15.1, while the mean SPQ-B score for the primary OCD group was 7.9. In prior unpublished research, we found that a score of 12 on the SPQ-B represented 1.5 SD above the mean and a significant proportion of subjects obtaining scores of 12 or greater met criteria for SPD. We also compared the neurocognitive profiles of the complete OCD sample and control subjects as a reference point for comparisons to prior neuropsychological studies. We completed four separate analyses:

1) we investigated differences between the complete OCD sample ($n = 25$) and community controls ($n = 11$);
2) we compared the performance patterns of primary OCD ($n = 15$) and control groups ($n = 11$);
3) we compared the performance patterns of OCD/SP ($n = 10$) and control groups ($n = 11$); and
4) we directly compared the neurocognitive profiles of primary OCD patients ($n = 15$) to the performance patterns of OCD/SP ($n = 10$) subjects.

## 3. Results

*3.1. Complete OCD sample*

Analysis of the performance of OCD patients ($n = 25$) and control subjects ($n = 11$) on neuropsychological tests revealed highly significant group differences on the Object Alternation Test and conflict blocks of the Stroop and Go-No Go tasks (putative orbitofrontal measures) (see Table 1). The mean number of trials to solve the Object Alternation Test was 7.7 for the control group, but was 37.1 for the OCD subjects (range 10–50). Group means for reaction time (RT) on the Stroop Color-Word Test also differed, with OCD subjects producing the slowest reaction times. While there was no significant difference between groups on the word naming block, the mean reaction time for color naming during the second block was greater among OCD subjects, suggesting that they experienced difficulty inhibiting the dominant response (i.e., word naming). Relative to control subjects, OCD patients exhibited slower reaction times for conflict blocks 2 & 3 on the Go/No-Go task, but not on the nonconflict block. This indicates that when required to inhibit a previously learned response pattern (conflict blocks 2 & 3), OCD patients displayed slower RTs. Contrary to expectation, OCD patients generated significantly fewer words (FAS Test) and alternate uses (DvT) (putative DLPF tasks) in comparison to control subjects (see Table 1). The latter finding is inconsistent with the contention that a select deficit involving the orbitofrontal system underlies OCD. Rather, these findings suggest that deficits involving inhibitory control (presumably involving orbitofrontal cortex) and executive function (possibly reflecting DLPF dysfunction) are associated with OCD.

Distinct neurocognitive profiles emerged when we examined the impact of comorbid schizotypy on the neuropsychological test performance of OCD patients. As noted previously, we observed that OCD patients who did poorly on executive function tasks had high schizotypy scores. This prompted a separate analysis by OCD subtypes.

*3.2. Neurocognitive function in primary OCD*

We compared the test performance of primary OCD patients ($n = 15$) and community control subjects ($n = 11$). The term "primary OCD" was used to describe OCD patients presenting without pronounced schizotypal personality features. In these individuals,

Table 1
Neurocognitive Profile: OCD & Control–Mean

| | OCD | Control | t | p | es* |
|---|---|---|---|---|---|
| n | 25 | 11 | | | |
| Age | 39.4 | 35.4 | 0.810 | 0.424 | – |
| Education | 15.6 | 16.1 | −0.599 | 0.553 | – |
| OAT** | 37.1 | 7.7 | 5.99 | 0.001 | 0.62 |
| Stroop Color-Word Test*** | | | | | |
| Stroop Word-c | 591 ms | 487 ms | 1.58 | 0.122 | – |
| StroopWord-i | 575 ms | 506 ms | 1.61 | 0.115 | – |
| StroopColor-c | 886 ms | 685 ms | 2.89 | <0.007 | 0.17 |
| StroopColor-i | 985 ms | 792 ms | 3.34 | <0.002 | 0.22 |
| Go/No-Go Task | | | | | |
| Go/No-Go–1 | 329 ms | 294 ms | 1.84 | 0.074 | – |
| Go/No-Go–2 | 496 ms | 441 ms | 2.60 | <0.02 | 0.14 |
| Go/No-Go–3 | 499 ms | 452 ms | 2.75 | <0.009 | 0.16 |
| FAS Test | 37.1 | 50.0 | −2.93 | <0.006 | 0.18 |
| DvT | 6.4 | 8.2 | −2.09 | <0.05 | 0.09 |

Note. OAT = Object Alternation Test; Stroop = Stroop Color-Word Test (blocks 1 & 2 = word naming, blocks 3 & 4 = color naming), c = congruent, i = incongruent; Go/No-Go = Go/No Go Task (blocks 1, 2, 3); FAS = Controlled Word Fluency Test (FAS Test); DvT = Divergent Thinking Task; ms =milliseconds.

*ES = Effect Size (Omega$^2$).

**Ten OCD patients and eleven control subjects completed the OAT.

***One OCD patient did not complete the Stroop task.

scores on the SPQ-B did not differ significantly from scores obtained by community subjects (based on published norms). The primary OCD group comprised nine female and six male subjects and their ages ranged from 20 to 63 years ($M = 39.6$; $SD = 14.7$). The mean educational level of the primary OCD group was 15.9 years ($SD = 1.6$). Thirteen primary OCD subjects were right-handed and two subjects were left-handed as determined by self-report. Analysis revealed significant group differences on the Object Alternation Test and conflict blocks of the Stroop and Go/No-Go tasks, while group differences on the nonconflict blocks of the Stroop and Go/No-Go tasks were not statistically significant (see Table 2). Although OCD patients produced fewer words (FAS Test) and alternate uses (DvT), group differences were not statistically significant supporting the claim that orbitofrontal dysfunction, rather than a global frontal deficit, is associated with primary OCD.

### 3.3. *Neurocognitive function in OCD/schizotypal personality*

The OCD/SP group comprised six male and four female subjects and their ages ranged from 21 to 68 years ($M = 38.9$; $SD = 15.0$). The mean educational level of the OCD/SP group was 15.3 years ($SD = 1.7$). All OCD/SP subjects were right-handed as determined by self-report. The OCD/SP patients differed significantly from community control subjects on the conflict blocks of the Stroop and Go/No-Go tasks, although differences were not as robust as those displayed by primary OCD patients (see Table 3). OCD/SP subjects generated significantly fewer responses on the Controlled Word Fluency Test and on the Divergent Thinking Task in comparison to control subjects. Relative to controls, subjects with OCD/SP were more impaired on tasks considered sensitive to orbitofrontal dysfunction and showed greater neuropsychological deficits on tests of executive function. In addition, the OCD/SP group displayed significantly greater RTs on the non-conflict block of the Go/No-Go and non-significant trends on the non-conflict blocks of the Stroop task. Results may reflect a general reduction in information processing speed among OCD/SP subjects. The mean number of trials to solve the Object Alternation Test was 19.0 for the OCD/SP group ($n = 2$), but was 7.7 for the community control group. This difference was statistically significant ($p < 0.001$).

### 3.4. *Direct comparison of primary OCD & OCD/SP groups*

We directly compared the neurocognitive and clinical profiles of primary OCD patients ($n = 15$) to the performance patterns of OCD/SP subjects ($n = 10$). We administered additional tests of executive function to OCD subjects including the Porteus Maze Task and

Table 2
Neurocognitive profile: Primary OCD & Control–Mean

| | Primary OCD | Control | t | p | es* |
|---|---|---|---|---|---|
| n | 15 | 11 | | | |
| Age | 39.6 | 35.4 | 0.821 | 0.420 | – |
| Education | 15.9 | 16.1 | −0.201 | 0.843 | – |
| OAT** | 41.6 | 7.7 | 7.60 | 0.001 | 0.74 |
| Stroop Color-Word Test | | | | | |
| Stroop Word-c | 536 ms | 487 ms | 1.73 | 0.095 | – |
| StroopWord-i | 550 ms | 506 ms | 1.19 | 0.243 | – |
| StroopColor-c | 924 ms | 685 ms | 3.23 | <0.004 | 0.27 |
| StroopColor-i | 993 ms | 792 ms | 3.07 | <0.005 | 0.24 |
| Go/No-Go Task | | | | | |
| Go/No-Go–1 | 316 ms | 294 ms | 1.49 | 0.150 | – |
| Go/No-Go–2 | 505 ms | 441 ms | 3.08 | <0.005 | 0.25 |
| Go/No-Go–3 | 497 ms | 452 ms | 3.00 | <0.006 | 0.23 |
| FAS Test | 41.2 | 50.0 | −1.89 | 0.07 | – |
| DvT | 7.1 | 8.2 | −1.22 | 0.232 | – |

Note. OAT = Object Alternation Test; Stroop = Stroop Color-Word Test (blocks 1 & 2 = word naming, blocks 3 & 4 = color naming), c = congruent, i = incongruent; Go/No-Go = Go/No Go Task (blocks 1, 2, 3); FAS = Controlled Word Fluency Test (FAS Test); DvT = Divergent Thinking Task; ms = milliseconds.

*ES = Effect Size (Omega$^2$).

**Eight primary OCD patients and eleven control subjects completed the OAT.

the Trail-Making Test (Parts A & B). In addition, OCD subjects completed the Frontal Lobe Personality Scale, the Limbic System Checklist-33 (LSCL-33), and the Schizotypal Personality Questionnaire-B (SPQ-B). We did not administer these additional measures to community control subjects. As noted previously, after administering the Object Alternation Test to 10 OCD patients and community control subjects ($n = 11$), we modified the task, based on the suggestion of Deacon (personal communication, 1999). We administered the OAT-MV to seven primary OCD patients and eight OCD/SP subjects.

As anticipated, primary OCD and OCD/SP subjects did not display significant group differences on tasks considered sensitive to orbitofrontal dysfunction (i.e., OAT-MV, and conflict blocks of the Stroop and Go/No-Go tasks). However, OCD/SP subjects showed greater neuropsychological deficits on tests of executive function (FAS Test & Porteus Maze RT) and scored significantly higher on a measure of temporolimbic dysfunction (the LSCL-33) in comparison to primary OCD patients. Analysis of primary OCD and OCD/SP subjects' scores on the Limbic System Checklist-33 (LSCL-33) revealed striking group differences. Mean score on the LSCL-33 for the OCD/SP group was 52.8, but was 25.2 for the primary OCD sample. Moreover, OCD/SP subjects achieved significantly higher scores on the Frontal Lobe Personality Scale. Although OCD/SP subjects exhibited slower reaction times on the Trail-Making Tests and produced fewer alternate uses (DvT), group differences did not achieve statistical significance. However, these non-significant trends were in the expected direction (see Table 4).

### 3.5. *Medication effects*

Almost half (48%) of the OCD subjects were undergoing pharmacotherapy at the time of testing (primarily SSRIs or clomipramine). None of the OCD subjects were receiving neuroleptics or atypical antipsychotics. To address the issue of medication effects on neuropsychological test performance, we compared the neurocognitive profiles of medicated and unmedicated OCD patients. The clinical and neurocognitive profiles of medicated and unmedicated OCD subjects were remarkably similar. Groups did not differ significantly on the Object Alternation Tests ($ps > 0.65$), Stroop conflict blocks (color-naming) ($ps > 0.65$), Go/No-Go task ($ps > 0.54$), Controlled Word Fluency Test ($p > 0.18$), Divergent Thinking Task ($p > 0.48$), Porteus Maze Task ($p > 0.18$), Trails (A) ($p > 0.18$), and Trails (B) ($p > 0.51$). However, medicated subjects displayed significantly greater RTs on the non-conflict block of the Stroop task, $t(23) = 2.116$, $p < 0.05$ and $t(23) = 2.619$, $p < 0.02$. None of the control subjects were taking psychotropic medications.

## 4. Discussion

The neurocognitive profiles of primary OCD and OCD/SP subjects may reflect abnormalities in distinct

Table 3
Neurocognitive profile: OCD/SP & Control–Mean

| | OCD/SP | Control | t | p | es* |
|---|---|---|---|---|---|
| n | 10 | 11 | | | |
| Age | 38.9 | 35.4 | 0.627 | 0.538 | – |
| Education | 15.3 | 16.1 | −0.877 | 0.392 | – |
| OAT** | 19.0 | 7.7 | 4.67 | <0.001 | 0.61 |
| Stroop Color-Word Test*** | | | | | |
| StroopWord-c | 682 ms | 487 ms | 1.97 | 0.064 | – |
| StroopWord-i | 618 ms | 506 ms | 1.79 | 0.089 | – |
| StroopColor-c | 824 ms | 685 ms | 1.88 | 0.076 | – |
| StroopColor-i | 970 ms | 792 ms | 3.27 | <0.004 | 0.33 |
| Go/No-Go Task | | | | | |
| Go/No-Go–1 | 348 ms | 294 ms | 2.17 | <0.05 | 0.15 |
| Go/No-Go–2 | 483 ms | 441 ms | 1.78 | 0.090 | – |
| Go/No-Go–3 | 501 ms | 452 ms | 2.19 | <0.05 | 0.15 |
| FAS Test | 31.0 | 50.0 | −4.14 | <0.001 | 0.43 |
| DvT | 5.4 | 8.2 | −2.36 | <0.03 | 0.18 |

Note: OAT = Object Alternation Test; Stroop = Stroop Color-Word Test (blocks 1 & 2 = word naming, blocks 3 & 4 = color naming), c = congruent, i = incongruent; Go/No-Go = Go/No Go Task (blocks 1, 2, 3); FAS = Controlled Word Fluency Test (FAS Test); DvT = Divergent Thinking Task; ms = milliseconds.

*ES = Effect Size (Omega$^2$).

**Two OCD/SP patients and eleven control subjects completed the OAT.

***One OCD/SP patient did not complete the Stroop task.

subdivisions of the prefrontal region. Primary OCD patients displayed impaired performance on measures sensitive to orbitofrontal dysfunction; however, they did not differ from control subjects on tests of executive function. OCD/SP subjects performed poorly on both orbitofrontal tasks and on measures of executive function. Several investigators documented executive function deficits among OCD patients, while other studies failed to find evidence of impaired executive function skills. We found that executive function deficits among OCD patients were associated with the presence of schizotypal features. Does the proportion of OCD subjects presenting with schizotypal personality features account, at least in part, for the conflicting findings in the neuropsychological literature?

Performance deficits on the Object Alternation Test may reflect an inability to effectively process feedback information regarding reward and punishment. Rolls [69] suggested that the orbitofrontal region determines the reinforcement value of stimuli. Poor performance on the Object Alternation Test among OCD subjects may reflect a deficit involving the inability to successfully employ reward and punishment cues to guide behavior. Impaired accuracy and RT differences on the Stroop and Go/No-Go tasks (conflict blocks) strongly correlate with Object Alternation Test performance in clinical and community samples suggesting that the orbitofrontal system is involved in the inhibition of a dominant response, which is consistent with its role in modifying behavior in response to changing contingencies. Rolls [69] observed, "(t)he neurophysiological and lesion evidence described suggests that one function implemented by the orbitofrontal cortex is rapid stimulus-reinforcement association learning, and the correction of these associations when reinforcement contingencies in the environment change." (p. 75).

In addition, there were clear-cut differences in the clinical presentation of the OCD subgroups. Both primary OCD and OCD/SP patients described obsessions and rituals that reflected a concern with threat and harm-avoidance. However, the OCD/SP subjects were also preoccupied with ordering rituals, striving for symmetry, and attaining perfection. The symptoms of primary OCD may represent compensatory strategies which serve to reduce the intense anxiety associated with orbitofrontal hypermetabolism. Similarly, the preoccupation with rules and organization, perfectionism, and inflexibility displayed by OCD/SP subjects may represent behavioral strategies which evolve in response to executive function deficits, possibly reflecting DLPF or mesial prefrontal dysfunction. Information obtained during post-testing interviews supported this claim. For example, primary OCD patients described checking behavior which was excessive and served to reduce intense, overwhelming anxiety. This behavior appeared to stem from an exaggerated fear of criticism and a heightened sense of personal responsibility. OCD/SP subjects also described checking be-

Table 4
Neurocognitive & clinical profiles: OCD/SP & Primary OCD

| | OCD/SP | Primary OCD | t | p | es* |
|---|---|---|---|---|---|
| n | 10 | 15 | | | |
| Age | 38.9 | 39.6 | −0.127 | 0.900 | – |
| Education | 15.3 | 15.9 | −0.923 | 0.366 | – |
| OAT-MV** | 48.0 | 45.8 | 1.05 | 0.309 | – |
| Stroop Color-Word Test*** | | | | | |
| StroopWord-c | 682 ms | 536 ms | 1.73 | 0.096 | – |
| StroopWord-i | 618 ms | 550 ms | 1.35 | 0.188 | – |
| StroopColor-c | 824 ms | 924 ms | −1.11 | 0.276 | – |
| StroopColor-i | 970 ms | 993 ms | −0.30 | 0.765 | – |
| Go/No-Go Task | | | | | |
| Go/No-Go–1 | 348 ms | 316 ms | 1.33 | 0.196 | – |
| Go/No-Go–2 | 483 ms | 505 ms | −0.77 | 0.445 | – |
| Go/No-Go–3 | 501 ms | 497 ms | 0.19 | 0.850 | – |
| FAS Test | 31.0 | 41.2 | −2.07 | <0.05 | 0.12 |
| DvT | 5.4 | 7.1 | −1.26 | 0.220 | – |
| Porteus-RT**** | 81.0s | 42.6s | 2.04 | <0.05 | 0.12 |
| Trail-Making Test | | | | | |
| Trails (A) | 43.5s | 34.0s | 1.47 | 0.154 | – |
| Trails (B) | 99.8s | 78.2s | 1.58 | 0.126 | – |
| FLPS-PV | 115.5 | 98.6 | 4.29 | <0.001 | 0.41 |
| SPQ-B | 15.1 | 7.9 | 6.34 | <0.001 | 0.61 |
| LSCL-33 | 52.8 | 25.2 | 4.75 | <0.001 | 0.46 |

Note. OAT-MV = Object Alternation Test-Modified Version; Stroop = Stroop Color-Word Test (blocks 1 & 2 = word naming, blocks 3 & 4 = color naming) , c = congruent, i = incongruent; Go/No-Go = Go/No Go Task (blocks 1, 2, 3); FAS = Controlled Word Fluency Test (FAS Test); DvT = Divergent Thinking Task; Porteus RT = Porteus Maze Task-reaction time; FLPS-PV = Frontal Lobe Personality Scale-Patient Version; SPQ-B = Schizotypal Personality Questionnaire-B; LSCL-33 = Limbic System Checklist-33; ms = milliseconds; s = seconds).

*ES = Effect Size (Omega$^2$).

**We administered the OAT-MV to seven primary OCD patients and eight OCD/SP subjects.

***One OCD/SP patient did not complete the Stroop task.

****One primary OCD patient did not complete the Porteus Maze Task.

havior which served to decrease acute anxiety. In addition, OCD/SP subjects described checking behavior which appeared to represent a compensatory response to working memory/executive function deficits and was accompanied by little or no anxiety.

The finding that OCD/SP subjects differed from primary OCD and controls on measures of executive function is particularly noteworthy. However, our findings must be interpreted with caution. One possible explanation is that primary OCD patients presented with a less severe form of OCD. Group differences may simply represent differences in the degree of symptom severity. It is possible that performance deficits on tests of frontal executive functioning displayed by OCD/SP subjects are due to differences in OCD symptom severity. Although groups met DSM-IV criteria for OCD (based on MINI scores), this measure does not assess symptom severity. However, our clinical impression, based on post-testing interviews, is that primary OCD and OCD/SP subjects did not differ significantly in the frequency and intensity of OC symptoms. Nevertheless, a systematic examination of the relationship between symptom severity and neuropsychological test performance is warranted.

Although we excluded potential participants if they met diagnostic criteria for major Axis-I disorders including major depressive disorder and bipolar disorder, it remains possible that the OCD groups may have differed in the intensity of subsyndromal depressive symptoms, which, in turn, had a deleterious effect on neurocognitive test performance. Since we did not assess general cognitive ability, it is also possible that performance deficits on prefrontal measures among OCD/SP subjects reflect "generalized" cognitive deficits, rather than a select deficit involving prefrontal executive function. However, primary OCD and OCD/SP groups did not differ on the Object Alternation Test-Modified Version, Stroop Color-Word Test, and the Go/No-Go Task. Moreover, all participants (both clinical and control) had attained a high level of formal education.

We are not suggesting that differences in the proportion of OCD patients presenting with schizotypal features entirely account for inconsistencies in the neuropsychological literature regarding executive function deficits. However, the relationship between patterns of Axis-II comorbidity among OCD patients and neuropsychological test performance merits further investigation. Our post-testing interviews revealed that most OCD/SP subjects had undergone traditional biobehavioral therapies (i.e., *in vivo* exposure and response prevention and/or SSRI pharmacotherapy), however, they had not received neuroleptic agents or atypical antipsychotics, or treatment addressing cognitive deficits. Clearly, an examination of the relation between executive function deficits and psychosocial adjustment among OCD/SP patients is warranted. Given that the presence of schizotypal personality features is a predictor of poor treatment response [49–51], this line of research may have significant treatment implications for OCD patients.

## Acknowledgements

The authors express appreciation to Kenneth Botelho (Noble Data, Inc., Norton, MA) for donating computer equipment and providing ongoing technical support.

The authors are grateful to Drs. Cary Savage, Michael Lyons, and Terrence Deacon for their helpful comments.

This research was supported by NIMH Grant 1 RO3 MH59255-01
